# Supplementary figures and images for: Construction of a novel five programmed cell death-related gene signature as a promising prognostic model for triple negative breast cancer
Source: PeerJ. 2025 Apr 28;13:e19359. doi: 10.7717/peerj.19359 (PMC12045267; doi:10.7717/peerj.19359)

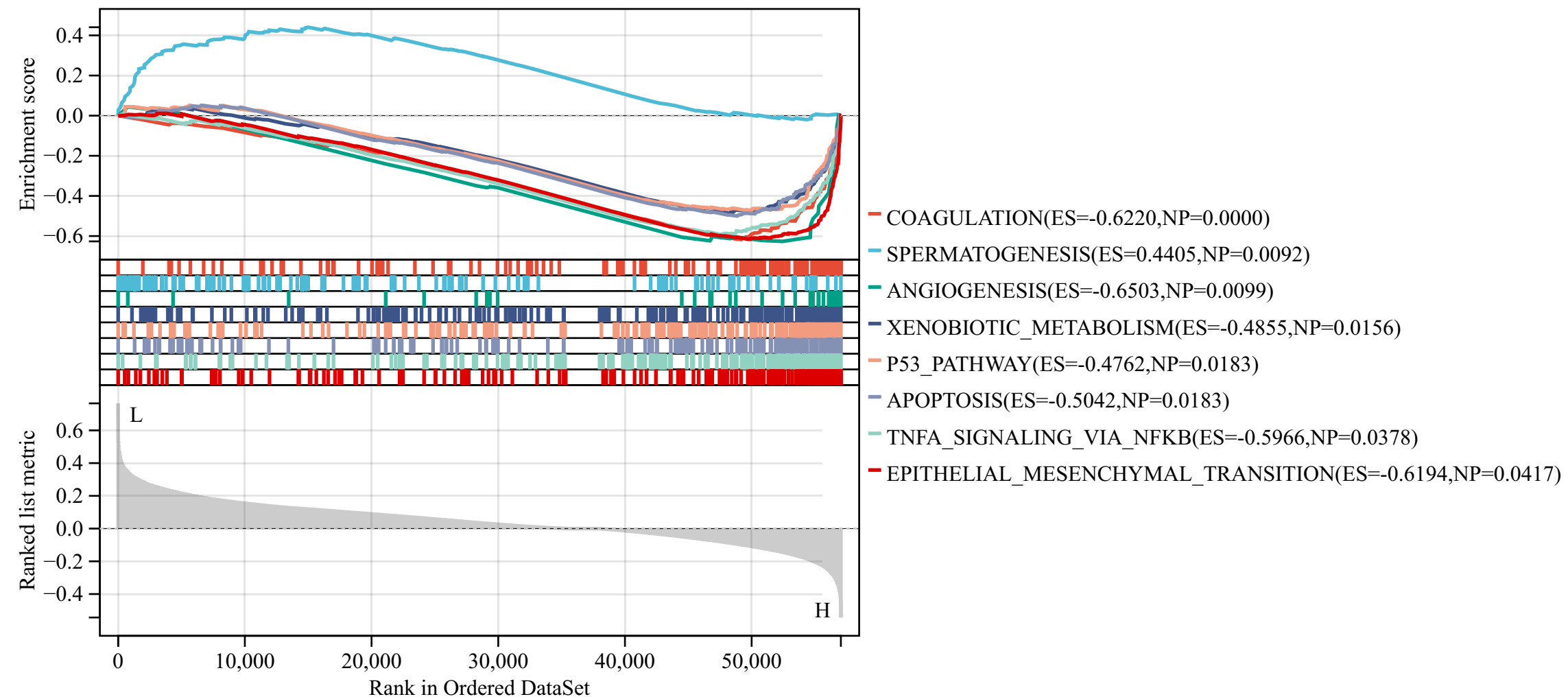

Supplement: Supplemental Information 4 [file peerj-13-19359-s004.pdf]
